# Effects of *Helicobacter pylori* adhesin HopQ binding to CEACAM receptors in the human stomach

**DOI:** 10.3389/fimmu.2023.1113478

**Published:** 2023-02-20

**Authors:** Quynh Anh Nguyen, Leonard Schmitt, Raquel Mejías-Luque, Markus Gerhard

**Affiliations:** Institute for Medical Microbiology, Immunology and Hygiene, School of Medicine, Technical University Munich, Munich, Germany

**Keywords:** *Helicobacter pylori*, CEACAMs, HopQ, CagA translocation, T4SS, NF-κB, gastric, inflammation

## Abstract

*Helicobacter pylori* has developed several strategies using its diverse virulence factors to trigger and, at the same time, limit the host’s inflammatory responses in order to establish a chronic infection in the human stomach. One of the virulence factors that has recently received more attention is a member of the *Helicobacter* outer membrane protein family, the adhesin HopQ, which binds to the human Carcinoembryonic Antigen-related Cell Adhesion Molecules (CEACAMs) on the host cell surface. The HopQ-CEACAM interaction facilitates the translocation of the cytotoxin-associated gene A (CagA), an important effector protein of *H. pylori*, into host cells *via* the Type IV secretion system (T4SS). Both the T4SS itself and CagA are important virulence factors that are linked to many aberrant host signaling cascades. In the last few years, many studies have emphasized the prerequisite role of the HopQ-CEACAM interaction not only for the adhesion of this pathogen to host cells but also for the regulation of cellular processes. This review summarizes recent findings about the structural characteristics of the HopQ-CEACAM complex and the consequences of this interaction in gastric epithelial cells as well as immune cells. Given that the upregulation of CEACAMs is associated with many *H. pylori*-induced gastric diseases including gastritis and gastric cancer, these data may enable us to better understand the mechanisms of *H. pylori*’s pathogenicity.

## 1 Introduction

Discovered in 1982 in the stomach of patients with gastritis and peptic ulceration (1), *Helicobacter pylori* – with its sophisticated mechanisms of pathogenesis – has gained much attention from many research groups over the past decades. Ten years later, in 1994, *H. pylori* was categorized by the World Health Organization as a class I carcinogen (2). This gram-negative, microaerophilic bacterium infects more than half of the world’s population at different rates depending on geographic location, with developing countries being the most affected regions with an *H. pylori* prevalence of up to over 80% (3, 4). According to many studies, the main routes of infection are thought to be oral-oral, fecal-oral, and gastro-oral *via* water or food consumption. Nevertheless, the exact mode of *H. pylori* transmission is not yet fully understood (5).

In 1992, Pelayo Correa demonstrated the progression of gastric pathologies that can develop as a result of a chronic *H. pylori* infection of the gastric mucosa. First, chronic gastritis and atrophy are induced, which can later progress to intestinal metaplasia, dysplasia, and finally gastric adenocarcinoma (6). While all *H. pylori* individuals develop chronic gastritis, gastric adenocarcinoma only occurs in 1-3% of cases (7). However, *H. pylori* was estimated to be responsible for 89% of non-cardia gastric cancer cases worldwide (8) and, according to GLOBOCAN 2020, gastric cancer is the fourth leading cause of cancer-related mortality for both sexes combined, accounting for around 1.08 million new cases and 769 000 deaths per year (9).

**Figure 1:**
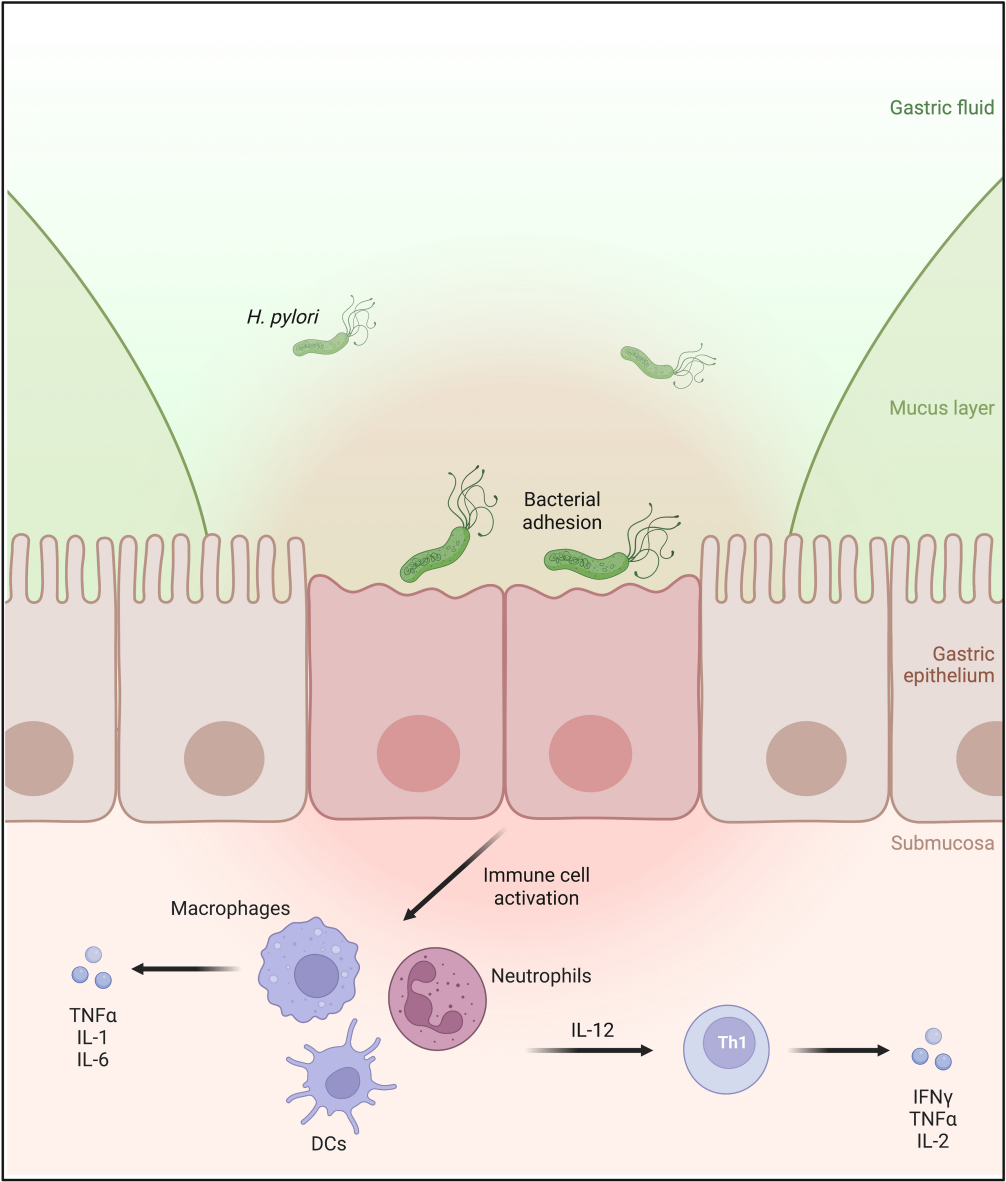
*H. pylori* infects the human stomach mucosa by binding to the apical side of the gastric epithelial cells. The infection causes immune cell infiltration of the gastric mucosa. Activated macrophages, dendritic cells, and neutrophils produce interleukin 12 (IL-12), which is involved in the differentiation of naïve T cells into Th1 lymphocytes, leading to the secretion of proinflammatory cytokines IFNγ and TNFα.

Over many thousands of years of colonizing the human stomach, *H. pylori* has diversified into numerous strains possessing various virulence factors (10). These have granted the bacterium the ability not only to directly affect the physiological and molecular processes of gastric epithelial cells but also to interact with the host’s immune cells to promote a strong inflammatory response (11) (**Figure 1**). One of *H. pylori*’s best-known virulence factors is the cytotoxin-associated gene A (CagA), which is injected by the bacterium into the host cells, leading to the activation of the host immune response and alteration of the host’s cellular processes (12–15). In addition, several bacterial adhesins of the *Helicobacter* outer membrane protein (Hop) family, including HopZ, BabA, SabA, and OipA, are also found to play a pivotal role in the colonization and pathogenesis of *H. pylori* (16, 17). Recently, another member of the Hop family, the HopQ adhesin, was reported not only to be involved in bacterial adhesion but also to be essential for the translocation of the effector protein CagA into the host cells by the Type IV secretion system (T4SS), which can aberrantly modify host cell signaling resulting in inflammatory responses. In 2016, HopQ was discovered by Javaheri et al. and Königer et al. to interact with the human Carcinoembryonic Antigen-related Cell Adhesion Molecules (CEACAMs) expressed on the host’s cell surface to facilitate the CagA translocation (14, 18). Since then, the HopQ-CEACAM interaction has been reported to act in a virulence-enhancing manner, possibly contributing to the development of many gastric pathologies. Due to the rising importance of the HopQ-CEACAM interaction, this minireview aims to summarize recent and novel findings of the molecular characterization, function, and consequence of this interaction.

## 2 *Helicobacter pylori* virulence factors: Type IV secretion system, CagA, HopQ

**Figure 2:**
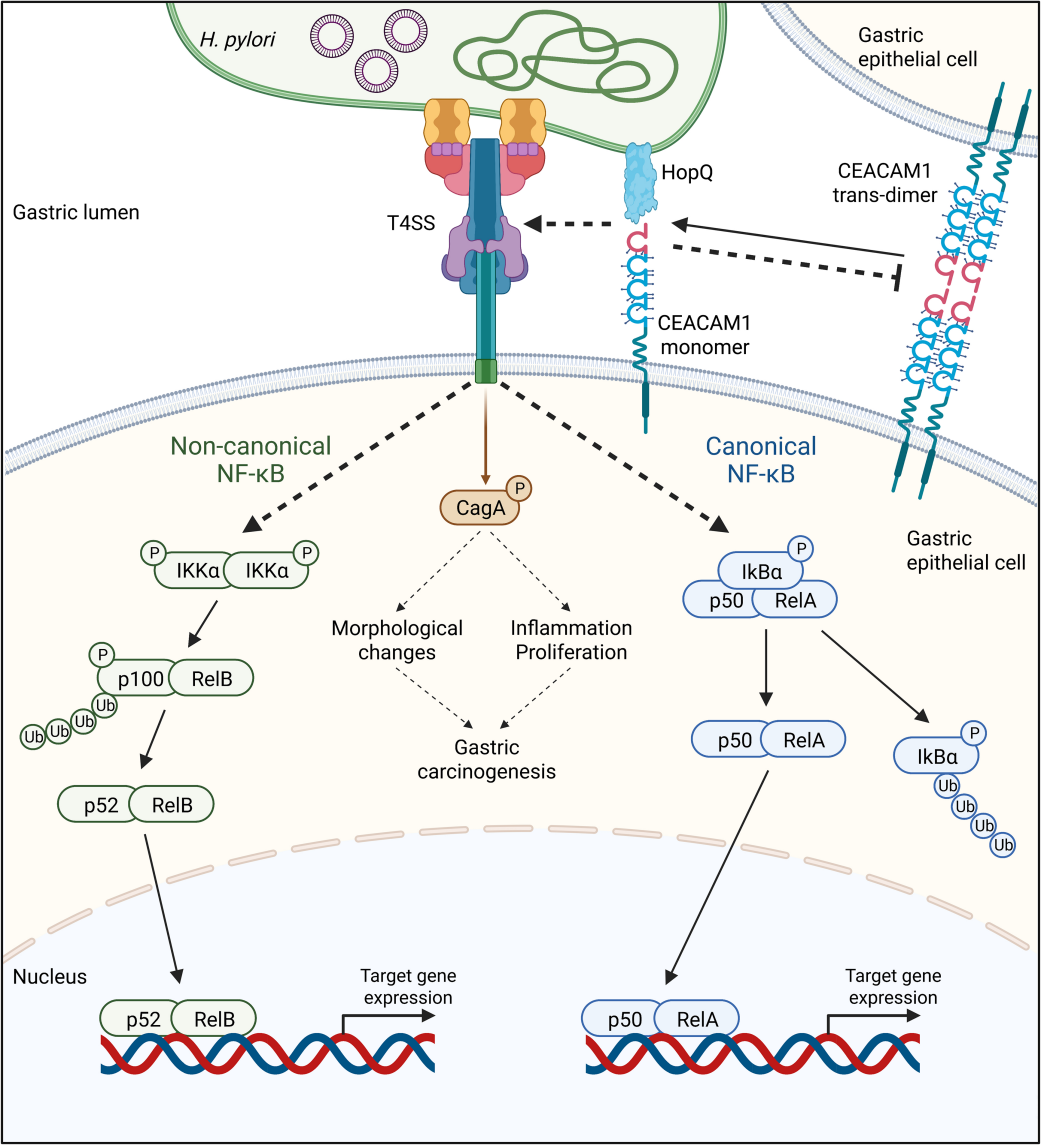
The adhesin HopQ binds to the N-terminal domain of CEACAM1, disrupting the trans-dimerization of the receptor. The HopQ-CEACAM1 interaction enables the translocation and phosphorylation of CagA and the activation of canonical and non-canonical NF-κB signaling in a Type IV secretion system (T4SS)-dependent fashion. Once activated, the transcription factor complexes p52/RelA of the canonical, as well as p52/RelB of the non-canonical NF-κB pathway, are translocated into the nucleus, activating the expression of target genes.

As a pathogen that colonizes and persists in the human stomach, *H. pylori* possesses numerous virulence factors. Among those, the T4SS, CagA, and HopQ play a critical role in the bacterium’s pathogenicity. HopQ binds human CEACAM receptors with high affinity and specificity, an interaction necessary for the translocation of CagA into infected cells *via* the T4SS (14, 18). Importantly, apart from enabling this translocation, the T4SS also seems to regulate the activation of the nuclear factor kappa-light-chain-enhancer of activated B cells (NF-κB) signaling pathway, resulting in the production of the pro-inflammatory cytokine IL-8 in host cells (19–22). This is in line with the fact that other bacterial factors such as peptidoglycan, bacterial nucleic acids, heptose-1,7-biphosphate (HBP), and ADP-glycero-β-D-manno-heptose, delivered by the T4SS into host cells, activate the NF-κB signaling (23–26). Similarly, HopQ binding to CEACAMs also acts as an essential regulator of the NF-κB pathway in a T4SS-dependent manner (21, 22) (**Figure 2**).

### 2.1 Type IV secretion system

Like several other bacteria, *H. pylori* possesses a T4SS, a pilus-like protein complex spanning both bacterial membranes and reaching into the extracellular space (27). The T4SS in *H. pylori* is encoded by the *cag* pathogenicity island (*cag*PAI), a DNA stretch of approximately 40 kb containing about 30 genes (28) originally derived from a bacteriophage. The core complex of the T4SS is located between *H. pylori*’s inner and outer membranes and comprises CagM, CagT, CagX, CagY, and Cag3 (29). It is connected to the extracellular pilus, made up of several proteins, including CagI, CagL, CagY, and CagA, which can target integrin α_5_β_1_ receptors on gastric epithelial cells (15). Once in contact with the host cell, the T4SS can fulfill its syringe-like function: the injection of *H. pylori* effector molecules into the host cell. Although also the translocation of peptidoglycan (23) and microbial DNA (24) through the T4SS have been discussed, the translocation of CagA (30) has received most attention (31). While the exact mechanism of this molecular injection remains unclear and goes beyond the scope of this review, its downstream effects in the host cells have been studied in depth.

### 2.2 Cytotoxin-associated gene A

**Figure 3:**
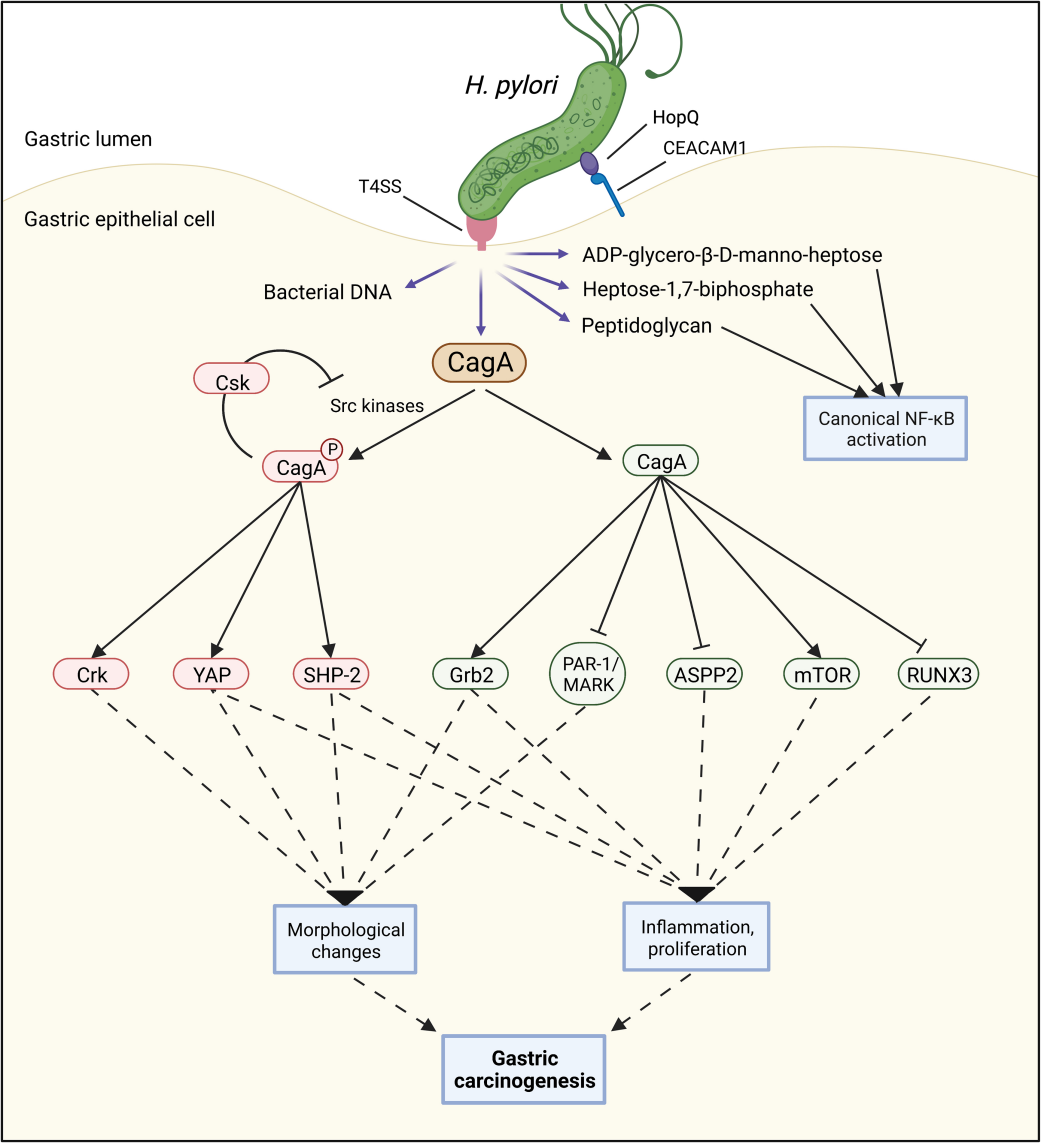
A functional Type 4 secretion system (T4SS) can facilitate the injection of several bacterial components such as bacterial DNA, peptidoglycan, heptose-1,7-biphosphate, ADP-glycero-β-D-manno-heptose, and CagA into the host cell. Once CagA is inside the host cell, both phosphorylated and non-phosphorylated CagA can interact with numerous proteins, contributing to several carcinogenic processes including morphological changes, inflammation, and cell proliferation.

CagA is a protein of 120–140 kDa (32) that is also encoded by the *cag*PAI (28, 32). After its translocation into the host cell cytoplasm *via* the T4SS, host kinases of the Src family phosphorylate tyrosine residues located within CagA’s Glu-Pro-Ile-Tyr-Ala (EPIYA) motifs (33). Both phosphorylated and non-phosphorylated CagA then interact with a multitude of host proteins, including the Src homology 2 (SH2)-containing tyrosine phosphatase 1 and 2 (SHP-1 and SHP-2) (34, 35), growth factor receptor-bound protein 2 (Grb2) (36), CT10 regulator of kinase (Crk) (37), and partitioning-defective 1 microtubule affinity-regulating kinase (PAR1/MARK) (38). This triggers numerous cellular pathways, leading to changes in cell morphology, signaling, and function. For instance, the dysregulation of the host cell’s actin cytoskeleton causes cells to elongate and adopt the aberrant so-called “hummingbird” phenotype (30). The cell’s polarity and interaction with adjacent cells are disrupted. At the same time, pro-proliferative, pro-inflammatory, and pro-oncogenic pathways are activated (39) (**Figure 3**).

In 1995, Blaser et al. reported that patients infected with CagA-proficient *H. pylori* strains had a higher risk of developing gastric cancer compared to patients infected with CagA-negative strains (40). These findings have since been confirmed and reviewed by Cover (41). It was not until 2008 that Ohnishi et al. were able to causally show that CagA, by aberrantly activating the oncogene SHP-2 in a phosphorylation-dependent manner, promotes gastric adenocarcinoma and MALT lymphoma development and can therefore be classified as an oncoprotein (42).

Many molecular mechanisms by which CagA contributes to gastric carcinogenesis are now known (43), with more being discovered every year, such as the activation of the oncogenic Yes-Associated Protein (YAP) pathway (44) or the mechanistic target of rapamycin complex 1 (mTORC1) pathway (45, 46). Furthermore, CagA inhibits several tumor suppressor proteins, including the runt-related transcription factor 3 (RUNX3) (47) and the apoptosis-stimulating protein of p53-2 (ASPP2) (48). In addition to these direct effects on cancer-promoting signaling pathways, CagA causes inflammation, which is known to be associated with cancer development (49).

CagA is highly imunogenic and induces an inflammatory response in the host organism that goes beyond the effects of *H. pylori* itself. As early as in 1997, Yamaoka showed a correlation between infection of CagA-proficient *H. pylori* strains and increased levels of the inflammatory cytokines IL-1β, IL-6, IL-8, and TNFα (50). Interestingly, at the same time, CagA seems to limit *H. pylori*’s inflammatory effects. *Via* an interaction with the C-terminal Src kinase (Csk), CagA inactivates the kinases responsible for its own phosphorylation. As a consequence, CagA-SHP-2 signaling and the subsequent inflammatory pathways are downregulated (51). CagA also downregulates cathepsin C (CtsC), impairing neutrophil activation (52), and induces tolerogenic, i.e., immune-suppressive, dendritic cells (DCs) (53, 54). By reducing the immune response against *H. pylori*, these mechanisms might support the bacterium’s viability and contribute to its long-term persistence in the human stomach.

In summary, CagA interferes with a multitude of cellular pathways, triggering a number of cellular responses that contribute to inflammation and gastric carcinogenesis. Hence, it is important to understand what enables its translocation *via* the T4SS.

### 2.3 HopQ

The *H. pylori* genome contains about 30 different *hop* genes, which encode outer membrane proteins (OMPs), some of these serving as bacterial adhesins, such as HopS (named BabA), HopZ, HopE or HopQ (55). This review will focus on HopQ. Two families of *hopQ* alleles exist: type I *hopQ* alleles are found more commonly in cag-positive *H. pylori* strains from patients with peptic ulcer disease, while type II *hopQ* alleles are found more commonly in cag-negative *H. pylori* strains from patients without ulcer disease (56).

Nowadays, it is known that HopQ is necessary for the translocation of CagA into host cells (57) and that this is achieved by the binding of HopQ to the human Carcinoembryonic Antigen-related Cell Adhesion Molecules (CEACAMs) 1, 3, 5, and 6 (14, 18). Interestingly, HopQ does not show homology to CEACAM-binding adhesins from other Gram-negative bacteria (14).

## 3 HopQ-CEACAM interaction

After its identification as a tumor-specific marker for colorectal cancer in 1965 by Gold and Freedman, the carcinoembryonic antigen (CEA), later classified as CEACAM5, was the first of a wide family of CEACAMs to be discovered as important regulators in carcinogenesis (58). They are mostly expressed on the surface of different cell types such as epithelial cells, endothelial cells, and immune cells (59). Besides their ability to mediate intercellular cell-cell adhesion and communication by forming homophilic and heterophilic cis- or trans-interactions on the cell surface (60, 61), CEACAMs are also important modulators for differentiation (62–64), proliferation (65), apoptosis (66), migration (67, 68), invasion (68), tumor development (69), and immune response (70, 71). Indeed, deregulation of CEACAMs was observed in many cancers such as of CEACAM6 in childhood acute lymphoblastic leukemias and early colorectal adenomas, or of CEACAM1 in colorectal and prostate cancer, as well as in melanoma (72–76). CEACAM1, -5, and -6 were also detected to be highly expressed in *H. pylori*-induced gastritis, precancerous lesions, and gastric cancer, while in a healthy stomach, they were only found at low expression levels, if at all (14, 22). In the human stomach, *H. pylori* was reported to induce the expression of CEACAMs at the apical side of epithelial cells, although the exact mechanism through which *H. pylori* modulates the expression of CEACAMs is still unclear. Then, *H. pylori* employs CEACAMs as receptors for its persistent binding.

Depending on the strain, *H. pylori* can bind specifically to CEACAM1, -3, -5, and -6 with different affinities, but no binding of the bacterium to CEACAM4, -7, or -8 could be observed (14). Importantly, both Javaheri et al. and Königer et al. showed that the N-terminal domain of CEACAM1 or CEACAM5 was the binding site for *H. pylori* HopQ. The binding of the bacterium is highly specific to human CEACAMs since no interaction with murine, bovine, or canine CEACAMs was observed. Interestingly, none of *H. pylori*’s known adhesins such as BabA, SabA, HopZ, and AlpA/B was involved in the binding of the bacterium to CEACAM1 and CEACAM5, but the outer membrane HopQ, which hence is the *bona fide* adhesin interacting with these receptors (14, 18). Indeed, the HopQ-deficient *H. pylori* strain P12Δ*hopQ* was incapable of binding to CEACAM1 and CEACAM5, and gastric cancer cells MKN28 lacking CEACAM did not bind recombinant HopQ (14). The bacterial adhesin was determined to form a strong, dose-dependent complex with CEACAM1 with a 1:1 stoichiometry in solution and in crystals, and a dissociation constant of K_D_ = 296 ± 40 nM (14, 77, 78).

**Figure 4:**
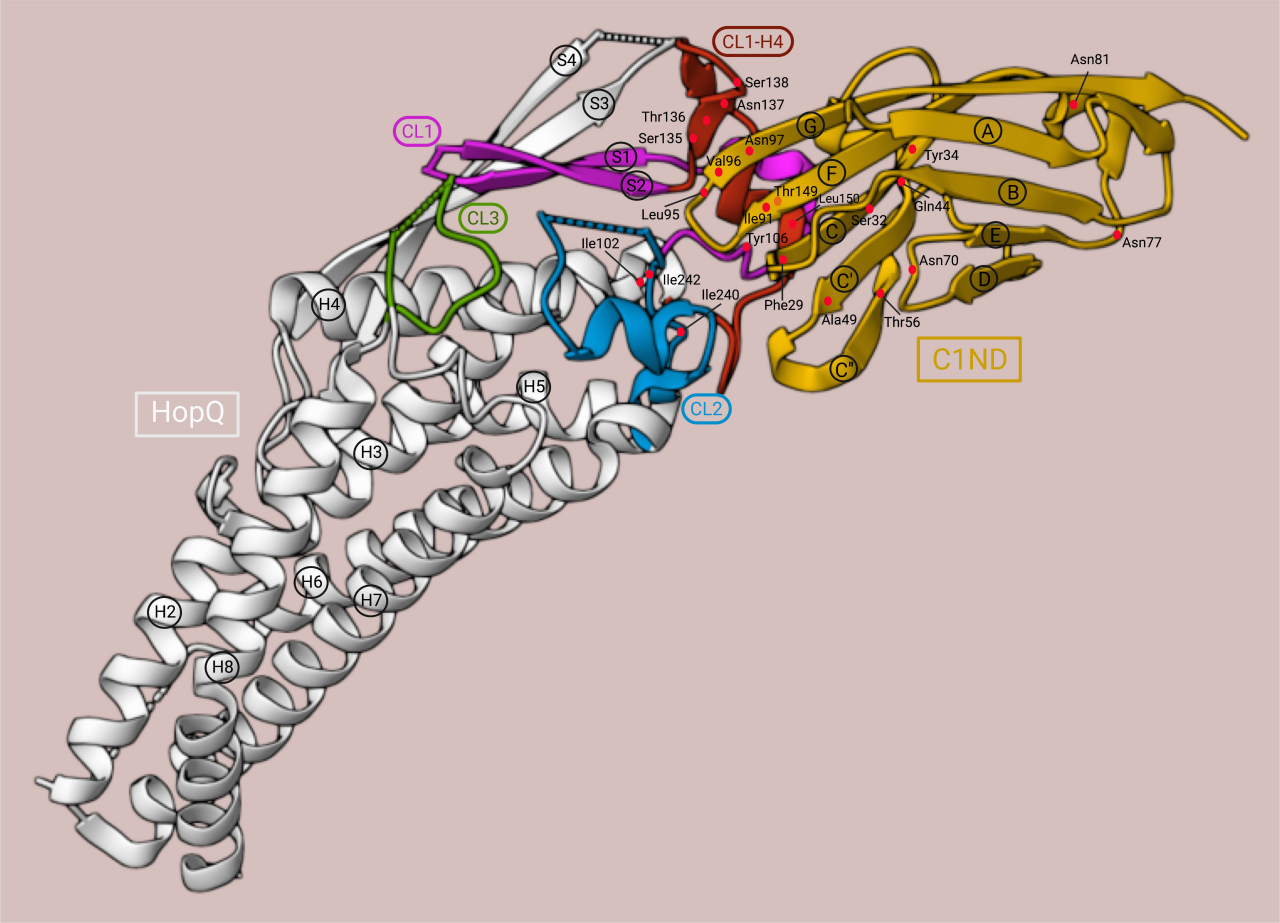
3D structure of the HopQ^AD-I^-C1ND interaction. C1ND, yellow; HopQ^AD-I^, white; CL1, magenta; CL1-H4, red; CL2, blue; CL3, green. The residues are depicted as red dots. This illustration was adapted from the crystal structure representation generated by Moonens et al. (78) using RCSB Protein Data Bank (79) (PDB ID: 6GBG, www.rcsb.org) and Mol* (80) (www.molstar.org).

Recently, the X-ray structure of the extracellular domain of HopQ type I (HopQ^AD-I^), which provides the interaction surface with the N-terminal domain of human CEACAM1 (C1ND), was revealed. A conserved 3 + 4-helix bundle topology with four β-strands (S1, S2, S3, and S4) and three disulfide-clasped loops (CL1, CL2, and CL3) was demonstrated as a high-resolution structure, of which each element was proven to contribute to the stability of the HopQ-CEACAM1 interaction (14, 78) (**Figure 4**). Two of the β-strands, S3 and S4, form the insertion domain (HopQ-ID), which contains binding sites for glycans. HopQ-ID was reported by Javaheri et al. to be crucial for the binding of HopQ to C1ND since the absence of this domain caused a tenfold decrease in binding affinity. However, the insertion domain was later shown to be located quite distantly from the binding interface and to contribute only indirectly to the C1ND binding by interacting with S1 and S2 to support the ordering of the CL1 and CL1-H4 (the loop connecting CL1 to Helix 4) loops into their binding conformation (77, 78). Clustering together, the CL1, CL1-H4, and CL2 loops establish protein-protein contact with the GFCC’C” interaction surface of the immunoglobulin-like (IgV) domain of C1ND in a glycan-independent manner (14, 78). Three N-linked glycosylation sites are anticipated to be present in the CEACAM1_IgV_ domain: Asn70^CEACAM1^, Asn77^CEACAM1^, and Asn81^CEACAM1^. They were found to point away from HopQ-ID, and thus have no significant impact on the interaction surface (77, 78). In fact, under enzymatic deglycosylation conditions, both CEACAM1_IgV_ and CEACAM1_IgC2_ exhibited no major difference in binding affinity compared to the glycosylated CEACAM1_IgV_ (14, 77).

In contrast to the mechanism observed for other bacterial proteins interacting with CEACAM receptors, HopQ-CEACAM1 binding is driven by a protein-protein interaction featuring three H-bonds between Gln44^CEACAM1^, Tyr34^CEACAM1^, and Ser32^CEACAM1^ with Thr149^HopQ^ in the CL1-H4 helix; five H-bonds between the strand G of C1ND and Ser135^HopQ^, Thr136^HopQ^, Asn137^HopQ^, and Ser138^HopQ^ in the CL1-H4 loop; and one H-bond between Thr56^CEACAM1^ in the strand C” and Tyr106^HopQ^ in the CL1. Additionally, the protein-protein contact is supported by hydrophobic bonds between the hydrophobic amino acids Phe29^CEACAM1^, Ile91^CEACAM1^, and Leu95^CEACAM1^ and the hydrophobic platform created by Ile102^HopQ^ in CL1-H3, Ile240^HopQ^, and Ile242^HopQ^ in CL2, and Leu150^HopQ^ in CL1-H4 (78). Notably, in a directed point mutation analysis, the mutation of Leu150^HopQ^ to Arg abolished the binding of HopQ^AD-I^ to C1ND completely. Moreover, the steric clashes in the interaction surface created by the mutation of Thr56^CEACAM1^ to Lys and Ala49^CEACAM1^ to Leu reduced the binding either only by 20% or in the same manner as the Δ*hopQ* mutant strain, respectively. This showed the importance of Leu150^HopQ^ and Ala49^CEACAM1^ for the interaction between C1ND and CL1 (78).

CL1, anchored by Cys103^HopQ^ and Cys132^HopQ^, was also proven to be a substantial component of the CEACAM1 binding site since Cys103Ser and Cys132Ser mutant strains did not show binding to CEACAM1 at the level observed for Δ*hopQ* mutant strains. Of note, the HopQ expression was maintained at the same level in the Cys103Ser mutant strain as in the wild-type strain, indicating that the lack of binding was not the result of a reduced expression of the adhesin but the loss of interaction with CEACAM1 on the interaction surface. In contrast, CL2 (anchored by Cys238^HopQ^ and Cys270^HopQ^) and CL3 (anchored by Cys362^HopQ^ and Cys385^HopQ^) mutants had no defects on CEACAM1 binding. As observed with the HopQ^AD-I^-CEACAM1 interaction, the loss of the disulfide bond of CL1 was also demonstrated to be crucial in HopQ^AD-I^ binding to CEACAM5 (81). CL1 and CL1-H4 loops were also reported to be disordered in the unbound HopQ^AD-I^; these loops were rearranged and became ordered only until they bound to C1ND through a coupled folding and binding mechanism (77, 78).

Using differential scanning fluorimetry, HopQ alone was measured to have a melting temperature (T_m_) of approximately 45°C between pH 5.5 and pH 7.0, and thus to be less stable than the HopQ-CEACAM1 complex, which not only had a T_m_ of about 50°C over the same pH range but also remained intact even at pH 3.0 (77). These results provide evidence, that, by binding to the N-terminal IgV domain of CEACAM1, HopQ type I enters a more stable structure than its unbound state. The X-ray structure of the extracellular domain of HopQ type II (HopQ^AD-II^) revealed that HopQ^AD-I^ and HopQ^AD-II^ target the same epitope on the GFCC’C” sheet of C1ND. Although HopQ^AD-II^ provides seven H-bonds less than HopQ^AD-I^ to the interaction surface with C1ND, it can bind C1ND with a sixfold higher affinity than that of the HopQ^AD-I^-C1ND interaction. Thus, HopQ^AD-II^ binding to CEACAM1 is rather hydrophobic and entropically driven, while the HopQ^AD-I^-C1ND interaction is enthalpically driven and entropically disfavored (78).

In C1ND, a β-sandwich fold with nine anti-parallel β-sheets is arranged in two opposing β-sheets: ABED, which is used by CEACAM1 to form cis-oligomerization on the same cell, and GFCC’C”, which is involved in the cross-cell trans-dimerization interface (82, 83). As discussed before, the non-glycosylated GFCC’C” surface is also in direct contact with HopQ^AD-I^, assembling H-bonds and hydrophobic bonds with CL1, CL1-H4 and CL2 (78). Indeed, a total of 26 residues were found to be shared by the interface that C1ND uses for HopQ^AD-I^ binding and trans-dimerization, including Phe29^CEACAM1^, Gln44^CEACAM1^, Ile91^CEACAM1^, Leu95^CEACAM1^, Val96^CEACAM1^, Asn97^CEACAM1^ (77). While the mutation of Phe29^CEACAM1^, Gln44^CEACAM1^, Val96^CEACAM1^, and especially Asn97^CEACAM1^ to Ala influenced the dimerization of CEACAM1 remarkably – in the case of Asn97Ala, dimeric CEACAM1 was even converted into monomers –, these mutations only caused a modest decrease in the binding energy with HopQ. This indicates that the binding energy with HopQ is distributed over many residues (77). Moreover, *H. pylori* can not only exploit the GFCC’C” interface of CEACAMs but also disrupt its trans-dimerization upon binding (77, 78). CEACAM1 was shown to be present as trans-dimer in solution, yet upon infection with *H. pylori* wild-type but not Δ*hopQ* mutant strain, the cross-linking efficiency of CEACAM1 is attenuated. Thus, in the presence of *H. pylori*, the equilibrium between dimeric and monomeric CEACAM1 is shifted to monomeric CEACAM1 to favor the formation of the HopQ-CEACAM1 complex (78).

## 4 Downstream effects of HopQ-CEACAM binding

An important consequence of the HopQ-CEACAM interaction is the T4SS-mediated CagA translocation and phosphorylation in the host cells. Nearly a decade ago, CagA translocation was for the first time reported to be HopQ-dependent by Belogolova et al. Out of 19 genes, which were found by screening transposon mutant library to alter T4SS-dependent activation of the NF-κB signaling pathway and secretion of IL-8, HopQ was identified as an important factor supporting the pro-inflammatory response and the CagA translocation (57). Nevertheless, it was not until 2016 that the binding partners of HopQ, CEACAMs, and the dependence of the CagA translocation and phosphorylation on the HopQ-CEACAM interaction were described by Javaheri et al. and Königer et al. This shed light on how *H. pylori* can utilize different virulence factors to interact with the host cells and thereby enhance its pathogenicity.

While the lack of HopQ leads to an alteration of *cag*PAI-dependent CagA translocation and a reduction of IL-8 secretion (14, 57), the impact on these events appeared to depend on different CEACAMs and cell lines. In gastric cell lines expressing high levels of CEACAM1, -5, and -6 such as AGS, KatoIII, and MKN45, CagA can be translocated into the host cells and successfully phosphorylated. Of note, the elevated expression levels of CEACAM5 and -6 were reported to foster CagA translocation even to a larger extent. In contrast, in cell lines expressing little or none of these CEACAMs, including MKN28, Hela, and HEK293, no CagA translocation could be observed (18). However, while the single knockdown of one of CEACAM1, -5, and -6 in AGS cells was not able to revoke CagA translocation, the simultaneous knockdown of all present CEACAMs in this cell line abolished CagA translocation to a similar extent as is observed when using the *H. pylori* Δ*hopQ* mutant strain for infection. This indicates a functional redundancy of CEACAMs in AGS cells. Moreover, the fact that the altered HopQ-CEACAM interaction only reduces the CagA translocation into AGS cells by 50% suggests that another receptor on AGS cells aside from CEACAMs or another *H. pylori* adhesin aside from HopQ might also be involved in CagA translocation (18). Conversely, in KatoIII cells, the triple knockdown of CEACAM1, -5, and -6, like the lack of HopQ, completely abrogated CagA translocation, indicating that in these cells, CagA translocation seems to be enabled mostly by the HopQ-CEACAM interaction (84).

Integrin is one of the alternative receptors expressed on gastric epithelial cells reported to be engaged for the T4SS-mediated CagA translocation (85). Previously, many studies on different cell lines have been focusing on the functional role of integrin in CagA translocation and signal transduction upon *H. pylori* binding. Like CEACAMs, integrin acts in different manners depending on the cell line. In AGS and KatoIII cells, the single knockout of integrin β_1_, the double knockout of integrin β_1_β_4_ and α_v_β_4_, and the triple knockout of all αβ integrins had no major impact on CagA translocation and the induction of IL-8 expression, in contrast to the abrogation of CEACAMs expression (84). Additionally, the absence of integrin-linked kinase (ILK), which interacts with the cytoplasmic domain of integrin β_1_ to mediate signaling from the extracellular matrix to the intracellular compartment (86), was also not essential for CagA translocation (21, 84). Thus, it was concluded that neither integrin interaction with the T4SS nor integrin signaling but HopQ-CEACAM interaction is required for CagA translocation into AGS and KatoIII cells (84). In another study using AZ-521 cells infected with a T4SS-defective strain, the overexpression of CEACAM1 and -5, but not CEACAM6 or integrin, was able to compensate for the insufficiency of the T4SS for CagA translocation (87). However, the fact that this cell line was reported to be a misidentified duodenal cancer cell line raises questions about the usefulness of using AZ-521 cells for studying the effects of *H. pylori* in the stomach.

Upon *H. pylori*-induced inflammation, several signaling pathways are activated including the canonical and non-canonical NF-κB (19–21, 88). While the activation of the canonical NF-κB pathway involves the phosphorylation and proteasomal degradation of IκBα, leading to the translocation of the heterodimer p50/RelA into the nucleus (89, 90), the activation of the non-canonical NF-κB pathway leads to the phosphorylation of IKKα, the phosphorylation and degradation of p100 to p52, and the translocation of the heterodimer p52/RelB into the nucleus (91–93) (**Figure 2**). Various studies have shown that both NF-κB pathways are mainly T4SS-dependent and CagA-independent (19–22). Indeed, in NCI-N87 and AGS cells infected with *H. pylori* lacking a functional T4SS, the phosphorylation and degradation of IκBα did not occur, in contrast to cells infected with the Δ*cagA* mutant strain (21). Similarly, the absence of CagE, a protein important for the T4SS pilus formation (94), but not CagA strongly reduced the processing of p100 to p52 in the non-canonical NF-κB pathway (22, 88).

Furthermore, the single knockdown, as well as the double knockdown of integrin α_5_ and/or β_1_, and the knockdown of ILK did not influence the activation of the canonical NF-κB signaling, showing that integrin-mediated pathway is dispensable for NF-κB activation during *H. pylori* infection (21, 84). In contrast, the HopQ-CEACAM interaction is a prerequisite for the activation of canonical and non-canonical NF-κB pathways. In gastric cells expressing high levels of CEACAMs, infection with the Δ*hopQ* mutant strain induced the activation of both pathways less effectively than wild-type *H. pylori* strains (21, 22, 95). However, NUGC-4 and SNU1 or Hela cells, expressing low levels of CEACAM1 or no CEACAMs, respectively, showed no significant changes in IL-8 secretion as well as the processing of p100 to p52 upon infection with HopQ-deficient *H. pylori*, indicating that efficient binding of HopQ to CEACAMs is necessary for the activation of these carcinogenic pathways (21, 22). Notably, a correlation between the upregulation of CEACAM1 and the activation of the non-canonical NF-κB pathway in *H. pylori*-induced gastritis, intestinal-type, and diffuse-type gastric tumors was also reported (22), strongly indicating a vital contribution of CEACAM1 to the pathogenic hallmarks of *H. pylori* infection.

Interestingly, the HopQ-CEACAM interaction is not only pivotal for the regulation of the epithelial cell response but also appears to modulate the immune response. During infection, *H. pylori* induces the infiltration of several immune cells including macrophages, DCs, neutrophils, and T cells (1, 96–99) (**Figure 1**). In human neutrophils expressing CEACAM1 and CEACAM6, CagA translocation and phosphorylation are facilitated effectively in a HopQ-dependent manner, whereas macrophages and DCs with low expression levels of these CEACAMs only allow low levels of CagA translocation and phosphorylation in a HopQ-independent manner (100). In addition, in murine neutrophils expressing human CEACAMs, thus exhibiting a functional HopQ-CEACAM interaction, infection with *H. pylori* infection increases the production of the proinflammatory chemokine MIP-1α compared to wild-type murine neutrophils. Conversely, upon infection, murine macrophages expressing human CEACAMs show significantly lower expression levels of CXCL1 and CCL2 than wild-type murine macrophages. This indicates the critical role of functional HopQ-CEACAM interaction in modulating chemokine secretion in different myeloid cells. In addition, by exploiting different CEACAMs, *H. pylori* can also regulate the oxidative burst of neutrophils and phagocytosis to support its intracellular survival in a HopQ-dependent fashion (100). The HopQ-CEACAM interaction can not only influence the activity of distinct myeloid cells in different ways but also affect the functions of natural killer (NK) cells and T cells. In activated CD4+ T cells expressing high levels of CEACAM1, IFNγ secretion is inhibited during infection with wild-type *H. pylori* in a HopQ-dependent manner. Similarly, the cytotoxic activity of activated CD8+ T cells and NK cells with high levels of CEACAM1 is also impeded during infection with the wild-type strain compared to Δ*hopQ* strain (101). These data suggest that *H. pylori*, and specifically HopQ, might be able to limit the inflammation caused by the bacterium’s infection, thereby possibly supporting lifelong persistence in the human stomach. The details of the bacterium’s immune evasion strategies have been reviewed before (102).

## 5 Discussion and outlook

Causing hundreds of thousands of premature deaths each year, *H. pylori*-associated diseases are a relevant factor for morbidity and mortality around the globe. However, given that about half of the world’s population is infected (3) and only relatively few individuals develop severe pathologies (103), it is clear that not all infections are equal. It is known that *H. pylori*’s oncoprotein CagA is one of the most important virulence factors contributing to the bacterium’s pathogenicity. The translocation of CagA *via* the T4SS and its subsequent interference with cellular processes are detrimental to gastric epithelial cells and explains a substantial part of *H. pylori*’s effects on inflammation and carcinogenesis (41, 104). CagA translocation requires the binding of *H. pylori*’s outer membrane protein HopQ to CEACAM receptors expressed on human gastric cells, and this interaction leads to different molecular changes, discussed in detail above. Although research is emerging on this matter, some questions are still unanswered.

As mentioned before, by binding to the trans-dimerization interface in the N-terminal domain of CEACAM1, HopQ can interfere with its monomer/dimer equilibrium (78). While many studies reported that the binding of SHP-1/2 to the tyrosine-phosphorylated Immunoreceptor tyrosine-based inhibitory motif (ITIM) of the CEACAM1-L variant supports the inhibitory effects of the ITIM on colon, prostate, and breast tumor cell growth (105–109), the role of ITIM and SHP-1/2 upon *H. pylori* infection of the gastric epithelial cells has not yet been studied intensively. Since the trans- or cis-dimerization of CEACAM1-L and the clustering of ITIM are required for SHP-1/2 to be sequestered to the ITIM domain (110), the disruption of the trans-dimerization of CEACAM1 in the presence of *H. pylori* HopQ leads to the release of SHP-1/2 from ITIM, possibly altering the downstream signaling transduction of CEACAM1, as proposed by Moonens et al. (78). However, ITIM seems to be dispensable for CagA translocation and phosphorylation, as the overexpression of CEACAM1-4S or CEACAM1-4L in MKN28 cells infected with *H. pylori* results in similar levels of phosphorylated CagA (78). Still, this needs to be confirmed in independent models and non-cancer cells. The fact that CagA also interacts with SHP-2 (34, 42) contributes even further to the complexity of this protein interaction network. Moreover, not only downstream but also upstream signaling of CEACAM1 upon *H. pylori* infection remains unknown. Considering the correlation between *H. pylori* infection and the upregulation of CEACAM1 in gastritis, pre-cancerous, and gastric cancer tissues (14, 22), the mechanisms through which *H. pylori* regulates CEACAM1 expression and signaling represent an interesting topic for future research.

Although integrin α_5_β_1_ was suggested by Kwok et al. to be a receptor required for T4SS to inject CagA into host cells (85), more recent studies have shown that integrins and their downstream signaling are not essential for the translocation and phosphorylation of CagA, in contrast to CEACAMs binding to HopQ (21, 84). It needs to be clarified whether the interaction between integrins and T4SS is compulsory for CagA translocation into host cells and, if at all, whether integrins and CEACAM receptors work in cooperation to support the tethering of T4SS onto host cells and the subsequent CagA translocation. It is also important to mention that the binding of *H. pylori* to CEACAMs during infection occurs mostly on the apical side of gastric epithelial cells (14, 18), while integrins, as receptors for cell-cell adhesion and cell attachment to the extracellular matrix (111, 112), are located at the basolateral side. The spatial difference between these two receptors raises questions about if and how *H. pylori* can interact with both receptors, and how they may interact with each other. In KatoIII cells, the triple knockout of CEACAM1, -5, and -6 was reported to have no significant influence on the intrinsic expression of α_v_ and β integrin. However, the triple knockdown of α_v_β_1_, α_v_β_4_, and β_1_β_4_ integrins in these cells resulted in a lower expression and recruitment of CEACAM5 to the binding surface with *H. pylori* compared to wild-types cells (84). Furthermore, Wessler and Backert suggested a new model, in which integrin-dependent T4SS activation for CagA translocation is facilitated through another virulence factor, the serine protease HtrA, which hijacks the tight junctions and adherence junctions between cells, enabling *H. pylori*’s transmigration to the basolateral side of polarized epithelial cells (113). Nevertheless, given the importance of CEACAM receptors, there are still unclarities about the role of integrin and the correlation between this receptor and CEACAMs in CagA translocation.

A recent study showed that recombinant HopQ coupled with fluorochromes was able to detect CEACAM-expressing colorectal tumors and metastases in a mouse model (114), hinting that this interaction might be exploitable for diagnostic purposes. Apart from that, impairing the HopQ-CEACAM interaction might represent a new approach for *H. pylori* prevention and treatment. Besides hygiene measures, no effective *H. pylori* prevention strategies exist. Moreover, while antibiotic treatment is still mostly efficacious, it has several limitations including costs, low compliance due to side effects and relatively long duration (10-14 days according to recent guidelines), and, most importantly, the risk of antimicrobial resistance. The inhibition of CEACAMs binding to *H. pylori*’s HopQ might impair the mechanism which makes the infection so hazardous. This could be achieved either by inhibiting the upregulation of CEACAMs, or by blocking the interaction of *H. pylori* to CEACAM receptors expressed on the host’s cell surface with, for example, competitive CEACAM-binders. Another strategy is to target the essential structural components of the HopQ-CEACAM complex. This offers new possibilities for prevention and treatment options targeted especially at those *H. pylori* infections with the greatest risk for gastric pathologies.

## Author contributions

QN and LS wrote the manuscript and conducted experimental work on the topic. RM-L and MG supervised the work and revised the manuscript. All authors contributed to the article and approved the submitted version.
